# Assessing the contribution of mild high-altitude exposure to obstructive sleep apnea-hypopnea syndrome comorbidities

**DOI:** 10.3389/fneur.2023.1191233

**Published:** 2024-01-08

**Authors:** Lijuan Hao, Kangkang Peng, Qi Bian, Suting Guo, Chengmin Duan, Lei Feng, Zhenguo Chen, Caiang Renzeng, Huaixia Pang, Zhen Ma

**Affiliations:** ^1^Department of Sleep Medicine, Qinghai Red Cross Hospital, Xining, China; ^2^Department of Otolaryngology, Graduate School of Qinghai University, Xining, China

**Keywords:** obstructive sleep apnea-hypopnea syndrome, apnea, oxygen desaturation index, hyponea treatment, mild high altitude

## Abstract

**Background:**

Obstructive sleep apnea-hypopnea syndrome (OSAHS) is a common sleep disorder. The lower atmospheric pressure and decreased oxygen levels of high-altitude areas can exacerbate the severity of OSAHS, but research into OSAHS in high-altitude areas remains limited. This study, from June 2015 to January 2020, involved 4,667 patients with suspected OSAHS and 38 healthy volunteers. The non-OSAHS group (AHI <5/h) had 395 patients, while the larger OSAHS group (AHI ≥5/h) comprised 4,272 patients. The significant size difference between the groups emphasized the study’s focus on OSAHS, using the non-OSAHS mainly for comparison.

**Methods:**

Sleep technicians monitored the OSAHS patient group overnight by polysomnography (PSG), the apnea-hypopnea index (AHI), the mean oxygen saturation (MSpO_2_), lowest oxygen saturation (LSpO_2_), the oxygen desaturation index (ODI) and the total sleep time with oxygen saturation less than 90% (TST-SpO_2_ <90%). Healthy volunteers self-monitored sleep patterns at home, using the CONTEC RS01 respiration sleep monitor with a wristwatch sleep apnea screen meter. The RSO1 wristwatch-style device has already been studied for consistency and sensitivity with the Alice-6 standard multi-lead sleep monitor and can be used for OSAHS screening in this region.

**Results:**

LSpO_2_ recordings from healthy volunteers (86.36 ± 3.57%) and non-OSAHS (AHI <5/h) cohort (78.59 ± 11.99%) were much lower than previously reported normal values. Regression analysis identified no correlations between AHI levels and MSpO_2_ or TST-SpO_2_ <90%, weak correlations between AHI levels and LSpO_2_ or MSpO_2_, and a strongly significant correlation between AHI levels and the ODI (*r* = 0.76, *p* < 0.05). The data also indicated that the appropriate clinical thresholds for OSAHS patients living at mild high altitude are classified as mild, moderate, or severe based on LSpO_2_ saturation criteria of 0.85–0.90, 0.65–0.84, or <0.65, respectively.

**Conclusion:**

The study findings suggest that individuals with an AHI score below 5 in OSAHS, who reside in high-altitude areas, also require closer monitoring due to the elevated risk of nocturnal hypoxia. Furthermore, the significant correlation between ODI values and the severity of OSAHS emphasizes the importance of considering treatment options. Additionally, the assessment of hypoxemia severity thresholds in OSAHS patients living in high-altitude regions provides valuable insights for refining diagnostic guidelines.

## 1 Introduction

Obstructive sleep apnea-hypopnea syndrome (OSAHS), also referred to as obstructive sleep apnea (OSA), is characterized by repetitive narrowing and collapse of the upper airway that can cause an individual to briefly stop breathing (apnea), or only take shallow breaths (hypopnea) while sleeping. As a result, oxygen levels in the blood are reduced and the sleep cycle is greatly disrupted (1, 2). According to the World Health Organization (WHO), an estimated 100 million people worldwide have OSAHS, with many cases that are undiagnosed and untreated (3). OSAHS is associated with a range of health problems, including cardiovascular disease, stroke, diabetes, and depression, among others. In severe cases, OSAHA contributes to severe comorbidities which increase mortality risk, such as cardiac arrest or respiratory failure (2, 3). The prevalence of OSAHS in the United States of America (USA) is 9%–24% for men and 4%–9% for women who are not obese (body mass index <30 kg/m^2^) and aged between 30 and 60 years, and it is estimated that approximately 25 million adults have some form of OSAHS, with the prevalence increasing with age (4). European countries have prevalence rates of 17%–30% in men and 5%–15% in women, with higher rates in older age groups (5), and it is estimated that millions of patients suffer from OSAHS in the Middle East and Arab countries (4). OSAHS is also common in Asian countries, particularly in China and India. One study estimated that the prevalence of OSAHS in China is approximately 10%, while another study has recorded that 13.7% of adults in India have OSAHS (3). Overall, OSAHS is a global health problem that affects a significant portion of the adult population, with a higher prevalence in older age groups and in individuals who are overweight or obese (2).

The classification of obstructive sleep apnea-hypopnea syndrome (OSAHS) uses the Apnea-Hypopnea Index (AHI), which counts apneic and hypopneic events per hour of sleep. OSAHS severity is based on AHI values: mild (5–14/h), moderate (15–30/h), and severe (>30/h) (6). Other key measures include mean and lowest oxygen saturation (MSaO_2_, LSaO_2_), oxygen desaturation index (ODI), and time with oxygen saturation below 90% (TST-SpO_2_ <90%). These assess oxygen desaturation severity during sleep and correlate with AHI (7–9). Higher AHI often means lower MSaO_2_ and LSaO_2_, higher TST-SpO_2_ <90%, and higher ODI, indicating more severe OSAHS (7, 10). However, there are currently few studies exploring these metrics in high-altitude areas, especially in mainland China. This study aims to investigate this and provide updated information.

Evidence indicates that the incidence of OSAHS is higher in high-altitude areas than in non-plateau areas (11, 12), which may be explained by several factors, such as lower oxygen levels, colder temperatures, and in response to ventilatory acclimatization to hypoxia at mild high altitude. Moreover, at mild high altitude, the reduced atmospheric pressure and lower oxygen levels can exacerbate the symptoms of OSAHS (11), Decreased oxygen saturation during sleep may increase the workload on respiratory muscles, potentially complicating the ability to keep the airway open, which is a central issue of OSAHS (13). Notably, some high-altitude areas may have limited medical resources, which could lead to the under-diagnosis and under-treatment of OSAHS (14, 15). The aim of this paper is to investigate the impact of high-altitude living on obstructive sleep apnea-hypopnea syndrome (OSAHS) and provide valuable insights for refining diagnostic guidelines.

## 2 Materials and methods

### 2.1 Subjects

Between June 2015 and January 2020, this study included 4,667 patients admitted to the Qinghai Red Cross Hospital (Qinghai, China) with suspected OSAHS. A substantial number of these patients presented clinical symptoms, often brought to their attention by family members or dormitory mates who observed severe snoring. Additionally, the study involved 38 healthy volunteers, primarily comprising hospital staff and their family members who did not exhibit clinical symptoms of OSAHS. The diagnosis and severity of OSAHS was based upon the AHI as either non-OSAHS (AHI <5/h; *n* = 395) or OSAHS (AHI >5/h; *n* = 4,272). The inclusion criteria were: (a) age between 12 and 70 years; (b) treatment-naïve, newly diagnosed patients; (c) no serious cardiovascular or cerebrovascular diseases; (d) no history of mental or neurological disorders. Exclusion criteria included: pregnancy or lactation, previous malignancy, mental illness, and any sleep disorder other than OSAHS. At the time of enrolment, each study participant had been living in the Qinghai province (with an average elevation of over 2,500 m) for over 1 year. The study was approved by the Qinghai Red Cross Hospital Institutional Review Board and it was conducted in compliance with national legislation and the Declaration of Helsinki guidelines. Neither the patients nor the general public were involved in the design or conduct of the study.

### 2.2 Respiration testing

Patients suspected of having OSAHS underwent overnight PSG using the Philips Alice-6 LDx system for at least 7 h. Simultaneous monitoring of respiratory parameters [AHI, oxygen desaturation index (ODI), mean oxygen saturation (MSpO_2_), lowest oxygen saturation (LSpO_2_), and the total sleep time with oxygen saturation less than 90% (TST-SpO_2_ <90%)] was conducted by sleep technicians. All participants arrived at the designated time to the sleep monitoring room at Qinghai Red Cross Hospital, where specialized sleep technicians attended to them. After a half-hour adaptation period, professional sleep technicians, working in two shifts, monitored the participants throughout the night. Machine data were reviewed the next morning, and the same specialized sleep technician manually assessed the images, exported the data, and recorded the results. Exclusion criteria for volunteers included no history of afternoon coffee consumption, heavy drinking, or the use of sedative, hypnotic, or muscle relaxant drugs, as well as the absence of a family history of sleep-related diseases. Healthy volunteers self-monitored sleep patterns at home, using the CONTEC RS01 Respiration Sleep Monitor with a wrist watch sleep apnea screen meter (Contec Medical Systems Co., Ltd.). RS01 monitoring results were collected and analyzed by the same group of sleep technicians on the second day. The sensitivity and accuracy of respiratory monitoring by RS01 is reportedly consistent with that of Alice-6 (16). All sleep monitoring results were analyzed and interpreted by two experienced professional sleep technicians.

### 2.3 Statistical analysis

Statistical analysis was conducted using the SPSS Statistics 19.0 software package (IBM Corporation, NY, United States). Receiver operating characteristic curve (ROC) analysis was performed using R (R 3.4.0 for Windows). Descriptive statistics for the data were presented as mean ± standard deviation (SD). Between-group differences were assessed using student’s *t*-tests for variance, while one-way analysis of variance (ANOVA) was employed to compare means among two or more groups. Partial correlation and linear regression models were utilized to control for covariates. The *p*-values were calculated to determine the significance of these relationships. It is important to note that we have followed a convention to underline the statistically significant values in our tables, denoting those with *p*-values less than 0.05.

## 3 Results

### 3.1 Patients living in mild high altitudes with AHI <5/h should not be considered “non-urgent”

**Figure 1:**
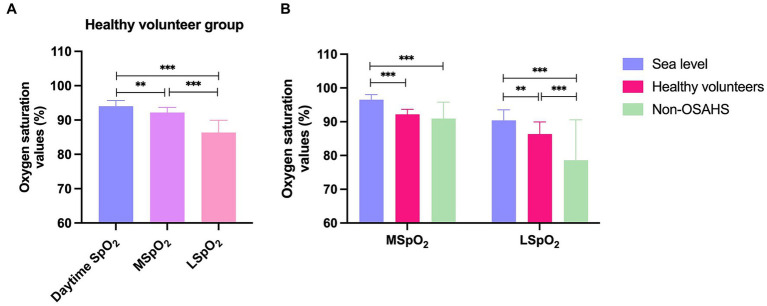
The oxygen saturation values defined as normal in previous publications, with the means for the healthy volunteers and non-OSAHS group in this study. **(A)** The oxygen saturation values of the healthy volunteer group. **(B)** The mean and lowest oxygen saturation values among the sea level, healthy volunteer, and non-OSAHS groups. SpO_2_ = oxygen saturation, how much oxygen the blood is carrying as a percentage of the maximum it can carry. MSpO_2_ = mean oxygen saturation, the average level of oxygen saturation during a specified period; LSpO_2_ = lowest oxygen saturation, the lowest recorded oxygen saturation level during the observation period, indicating the severity of respiratory disruptions. Descriptive statistics for the data were presented as mean ± standard deviation (SD) and the SD values were visually represented using error bars. Statistically significant results are denoted with an asterisk(*), with *p < 0.05, **p < 0.01, and ***p < 0.001 indicating the significance levels.

As shown in Table 1, the healthy volunteer group had a mean daytime SpO_2_ of 94.03 ± 1.66, MSpO_2_ of 92.19 ± 1.49 and LSpO_2_ of 86.36 ± 3.57, indicating that the average blood oxygen saturation during sleep at night was significantly lower than that during the daytime (*p* < 0.05) (Figure 1A). The much lower LSpO_2_ value compared with daytime SpO_2_ and MSpO_2_ values in the healthy volunteer group (*p* < 0.001) (Figure 1A) indicates that hypoxic sleep at night can occur even among healthy persons living at mild high altitudes. Previous research has reported that a normal reading for daytime SpO_2_ is typically between 96% and 99% (17, 18). At 1,600 meters altitude, oxygen saturation should be above 92% (19). In this study, the MSpO_2_ of the healthy volunteer group was significantly lower than people living at sea level (*p* < 0.001) (Figure 1B). Importantly, we found the daytime SpO_2_ values of non-OSAHS were 91.01 ± 6.10, which was lower than the expected 92% saturation at an altitude of 1,600 meters (Table 1 and Figure 1B). The normal values of LSpO_2_ have been known as 90.4 ± 3.1%, and MSpO_2_ have been known as 96.5 ± 1.5% (20). This study identified much lower LSpO_2_ values recorded from the healthy volunteers (86.36 ± 3.57%) and non-OHSAS (78.59 ± 11.99%) groups compared with normal values in previous reports (*p* < 0.001) (Figure 1B) (20). We also found significantly lower LSpO_2_ values in the non-OSAHS group (78.59 ± 11.99) compared with those of the healthy volunteers (86.36 ± 3.57) (*p* < 0.001) (Table 1 and Figure 1B), with the non-OSAHS group exhibiting excessive daytime sleepiness, poor concentration, memory problems and mood changes (data not shown).

**Table 1:** Oxygen saturation values for the healthy volunteers and the non-OSAHS and OSAHS groups.

Variable	Healthy volunteers (*N* = 38)	Non-OSAHS (*N* = 395)	OSAHS (*N* = 4,272)	*p*-value
Daytime SpO_2_	94.03 ± 1.66	91.01 ± 6.10	89.28 ± 4.84	<0.01^*^
MSpO_2_	92.19 ± 1.49	90.93 ± 4.87	86.23 ± 7.25	0.02^*^
LSpO_2_	86.36 ± 3.57	78.59 ± 11.99	65.57 ± 15.67	<0.01^*^
TST-SpO_2_ <90%	26.17 ± 28.12	55.34 ± 114.93	203.80 ± 156.16	<0.01^*^
ODI	3.12 ± 2.59	6.60 ± 9.19	48.43 ± 32.13	<0.01^*^

An AHI value of <5/h was defined as non-OHSAS; AHI ≥5/h was defined as OSAHS. ^*^A *p*-value of <0.05 was considered to be statistically significant by one-way ANOVA. OSAHS, obstructive sleep apnea-hypopnea syndrome; SpO_2_, oxygen saturation; LSpO_2_, lowest oxygen saturation; MSpO_2_, mean oxygen saturation; TST-SpO_2_ <90%, the total sleep time with oxygen saturation less than 90%; ODI, oxygen desaturation index.

### 3.2 ODI values differentiate OSAHS patients with relatively high accuracy

**Figure 2:**
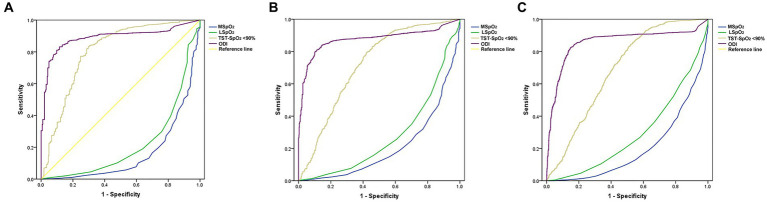
Receiver operating characteristic curve (ROC) analysis of MSpO_2_, LSpO_2_, TST-SpO_2_ <90% and ODI values at (A) AHI ≥ 5 to < 15/h, (B) AHI ≥ 15 to < 30/h, and (C) AHI ≥ 30/h.

**Figure 3:**
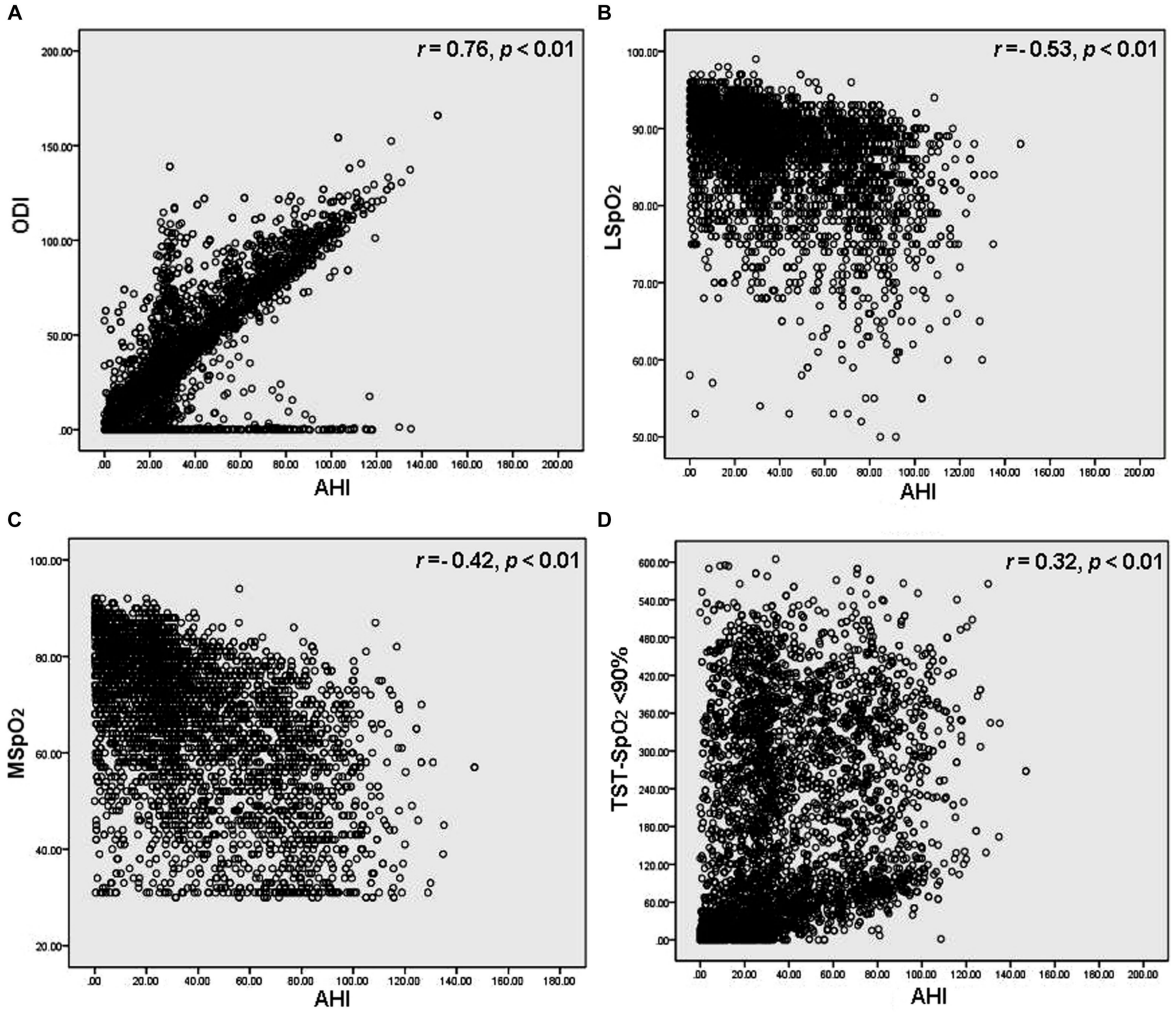
Regression analysis of correlations between AHI levels and **(A)** ODI, **(B)** LSpO_2_, **(C)** MSpO_2_ and **(D)** TST-SpO_2_ <90% values. Every data point corresponds to an individual participant.

After dividing the OSAHS cohort into 4 groups (AHI <5/h; AHI ≥5 to <15/h; AHI ≥15 to <30/h; and AHI ≥30/h), associations were analyzed between the AHI groups and LSpO_2_, MSpO_2_, TST-SpO_2_ <90%, and ODI values. Among people residing in high-altitude areas, LSpO_2_ levels were negatively associated with AHI values (*p* < 0.01), whereas the TST-SpO_2_ <90% and ODI values were positively associated with AHI values (*p* < 0.01) (Table 2). No associations were observed between MSpO_2_ and overall AHI values (*p* = 0.65), but MSpO_2_ was significantly decreased in the AHI ≥30/h group (Table 2). In a ROC analysis, the ODI was associated with the highest specificity and sensitivity values (Figure 2). The sensitivity and specificity of the ODI scores were 0.831 and 0.895, 0.810 and 0.887, and 0.825 and 0.857 for the AHI <5/h, AHI ≥5 to <15/h, AHI ≥15 to <30/h and AHI ≥30/h groups, respectively (Table 3). In linear regression analysis, a strongly significant correlation was identified between AHI levels and ODI values (*r* = 0.76, *p* < 0.01) (Figure 3A), weak correlations were observed between AHI levels and LSpO_2_ or MSpO_2_ values (*r* = 0.53 and 0.42, *p* < 0.01) (Figures 3B,C), while no correlation was observed between AHI levels and TST-SpO_2_ <90% values (*r* = 0.32, *p* < 0.01) (Figure 3D).

**Table 2:** Associations between LSpO_2_, MSpO_2_, TST-SpO_2_ <90%, and ODI values with AHI grades.

Variable	AHI <5/h (*N* = 395)	AHI ≥5 to <15/h (*N* = 657)	AHI ≥15 to <30/h (*N* = 1,245)	AHI ≥30/h (*N* = 2,370)	*p*-value
LSpO_2_	76.53 ± 12.78	74.86 ± 11.99	72.30 ± 11.58	59.50 ± 15.67	<0.01^*^
MSpO_2_	89.48 ± 9.67	89.36 ± 4.83	88.17 ± 6.03	84.37 ± 7.80	0.65
TST-SpO_2_ <90%	62.40 ± 117.54	131.42 ± 153.19	187.06 ± 160.31	232.35 ± 146.92	<0.01^*^
ODI	8.87 ± 10.48	15.70 ± 10.73	31.68 ± 20.39	66.59 ± 29.26	<0.01^*^

^*^A *p*-value of <0.05 was considered to be statistically significant by one-way ANOVA. OSAHS, obstructive sleep apnea-hypopnea syndrome; LSpO_2_, lowest oxygen saturation; MSpO_2_, mean oxygen saturation; TST-SpO_2_ <90%, the total sleep time with oxygen saturation less than 90%; ODI, oxygen desaturation index. AHI <5/h was defined as non-OSAHS and AHI ≥5/h was defined as OSAHS.

**Table 3:** ODI performance in ROC analysis: sensitivity and specificity across different AHI categories.

Variable	*N*	AUC	Cut-off points	Sensitivity	Specificity
AHI ≥5 to <15/h	657	0.889	12.45	83.1%	89.5%
AHI ≥15 to <30/h	1,245	0.875	21.90	81.0%	88.7%
AHI ≥30/h	2,370	0.855	37.05	82.5%	85.7%

ODI, oxygen desaturation index; AUC, area under the curve; OSAHS, obstructive sleep apnea-hypopnea syndrome; *N*, number; AHI, apnea-hypopnea index.

### 3.3 The relationships between OSAHS severity and different sleep stages

Average oxygen saturation differs during rapid eye movement (REM) and non-REM (NREM) sleep, according to the severity of sleep breathing disorders (21). In this study, we found that in the AHI <5/h cohort, MSpO_2_ values (90.41 ± 4.27) differed between wakefulness (WK) (91.18 ± 4.61) and REM states, respectively, (90.36 ± 5.11) (*p* < 0.01), but did not differ significantly between REM and NREM sleep (*p* = 0.16) (Table 4), whereas for all OSAHS patients, MSpO_2_ values gradually decreased from WK to NREM and then to REM sleep (*p* < 0.01) (Table 4). Previous research has reported that oxygen saturation in patients with mild or moderate OSAHS does not differ significantly between REM and NREM states (21). Our study identified significantly higher MSpO_2_ values in patients with mild or moderate OSAHS during REM sleep than during NREM sleep (*p* < 0.01 for both comparisons) (Table 4), these results were different with OSAHS patients not living at mild high altitude.

**Table 4:** MSpO_2_ values according to different sleep stages.

Variable	*N*	MSpO_2_ (WK)	MSpO_2_ (NREM)	MSpO_2_ (REM)	*p*1 value^a^	*p*2 value^b^	*p*3 value^c^
AHI <5/h	395	91.18 ± 4.61	90.41 ± 4.27	90.36 ± 5.11	<0.01	<0.01	=0.16
AHI ≥5 to <15/h	657	90.57 ± 4.66	89.49 ± 4.34	89.25 ± 5.38	<0.01	<0.01	<0.01
AHI ≥15 to <30/h	1,245	89.53 ± 4.07	87.94 ± 4.65	87.22 ± 5.86	<0.01	<0.01	<0.01
AHI ≥30/h	2,370	87.81 ± 5.15	84.53 ± 6.79	81.85 ± 9.17	<0.01	<0.01	<0.01

^*^A *p*-value of <0.05 was considered to be statistically significant by student’s *t*-test. MSpO_2_, mean oxygen saturation; *N*, number; AHI, apnea-hypopnea index; WK, wakefulness; NREM, non-rapid eye movement; REM, rapid eye movement.

a *p*1 = comparison between WK and NREM.

b *p*2 = comparison between WK and REM.

c *p*3 = comparison between NREM and REM.

### 3.4 Evaluating hypoxemia severity thresholds in OSAHS patients living in high-altitude areas

In China, two guidelines have been issued for OSAHS diagnosis and treatment: (guideline 1) diagnosis and treatment of obstructive sleep apnea hypopnea syndrome (22) and (guideline 2) the guideline for primary care of adult obstructive sleep apnea: practice version (2018) (23). One difference between these guidelines is the thresholds of LSpO_2_, which are given as 0.85–0.90, 0.65–0.85 or <0.65 as mild, moderate, or severe in guideline 1; and 0.85–0.90, 0.80–0.85 or <0.80 as mild, moderate, or severe in guideline 2 (Table 5). LSpO_2_ values have been used to assess hypoxemia severity (mild, moderate or severe hypoxemia) (24), with the different LSpO_2_ values affecting the treatment strategy. We therefore analyzed the clinical thresholds of AHI and LSpO_2_ by following these two guidelines. As shown in Table 5, when we classified our OSAHS cohort by guideline 2, more than 50% of the patients were found to have severe hypoxemia, even in the mild OSAHS cohort. When we used Spearman rank correlation analysis to measure the degrees of association between AHI and LSpO_2_ values in each guideline, the correlation coefficient *R*_s_ values were 0.495 and 0.361 for guidelines 1 and 2, respectively (Table 6). These data indicate that the appropriate clinical thresholds for assessing hypoxemia severity for OSAHS patients residing in high-altitude areas are values of 0.85–0.90, 0.65–0.85 or <0.65 graded as mild, moderate or severe hypoxemia, respectively.

**Table 5:** Comparisons of correlations between AHI grades and LSpO_2_ values for classifying patients by OSAHS severity by two different clinical guidelines issued in China.

Variable	Diagnosis and treatment of obstructive sleep apnea hypopnea syndrome (guideline 1)			Guideline for primary care of adult obstructive sleep apnea: practice version (2018) (guideline 2)		
	0.85 ≤LSpO_2_ <0.90	0.65 ≤LSpO_2_ <0.85	LSpO_2_ <0.65	0.85 ≤LSpO_2_ <0.90	0.80 ≤LSpO_2_ <0.85	LSpO_2_ <0.80
AHI ≥5 to <15/h	124 (18.87%)	385 (58.60%)	148 (22.53%)	124 (18.87%)	177 (26.94%)	356 (54.19%)
AHI ≥15 to <30/h	112 (9.00%)	804 (64.58%)	329 (26.43%)	112 (9.00%)	252 (20.24%)	881 (70.76%)
AHI ≥30/h	20 (0.84%)	946 (39.92%)	1,404 (59.24%)	20 (0.84%)	112 (4.73%)	2,238 (94.43%)

LSpO_2_, lowest oxygen saturation; AHI, apnea-hypopnea index.

**Table 6:** Spearman rank correlation coefficients (*R*_s_) and *p*-values of two OSAHS clinical guidelines issued in China.

Guidelines	*R*_s_	*p*-value^*^
Diagnosis and treatment of obstructive sleep apnea hypopnea syndrome (guideline 1)	0.495	0.00
Guideline for primary care of adult obstructive sleep apnea: practice version (2018) (guideline 2)	0.361	0.00

## 4 Discussion

OSAHS can impact significantly upon health and quality of life (1, 2). In high-altitude environments, the reduced atmospheric pressure and lower oxygen levels can exacerbate OSAHS symptoms, as the reduced oxygen levels can increase the workload on the respiratory muscles and make it more difficult to maintain an open airway during sleep (11, 12). In this research, we discovered a significant difference in LSpO_2_ (86.36 ± 3.57) compared to both daytime SpO_2_ (94.03 ± 1.66) and MSpO_2_ (92.19 ± 1.49) values within the healthy volunteer group (*p* < 0.001). This study highlights that even healthy persons residing in a high-attitude environment can experience night-time episodes of hypoxic sleep.

The AHI is a measure of the severity of sleep apnea, calculated as the number of apneic or hypopneic events per hour of sleep (6). An AHI value of less than 5 means that a person experiences fewer than 5 episodes of apnea (complete cessation of breathing) or hypopnea (partial obstruction of the airway) per hour of sleep (6). This is considered within the normal range and suggests that the person does not have significant sleep breathing disorders such as OSA. However, this study we identified much lower LSpO_2_ values recorded from the non-OHSAS (78.59 ± 11.99%) cohort (AHI <5/h) compared with normal values (90.4 ± 3.1%) in previous reports (*p* < 0.001) (20). We also found significantly lower LSpO_2_ values in the non-OSAHS group (78.59 ± 11.99) compared with those of the healthy volunteers (86.36 ± 3.57) (*p* < 0.001), with the non-OSAHS cohort presenting with excessive daytime sleepiness, poor concentration, memory problems, and mood changes. Notably, low LSpO_2_ levels are associated with an increased risk of cardiovascular and respiratory diseases. We therefore strongly suggest that for people living in high-altitude environments, AHI values of <5/h do not necessarily mean that the patient, who reside in high-altitude areas, should be considered “non-urgent” for treatment. This group should have medical care under the guidance of a healthcare professional.

High-altitude areas are typically associated with difficult terrains, extreme weather conditions and remote locations, making it challenging to access and transport medical supplies, equipment, and personnel. Moreover, the mild high altitude itself can pose health risks, such as the development of OSAHS (11–13). Several other measures, including SpO_2_ and ODI values, are important when evaluating the impact of OSAHS on a patient’s health (7–10). In this study, we analyzed associations between AHI levels and ODI, LSpO_2_, MSpO_2_, and TST-SpO_2_ <90% values by regression analysis. We found a strongly significant correlation between AHI levels and ODI (*r* = 0.76, *p* < 0.01), with the ODI differentiating levels of OSAHS severity with relatively high accuracy in patients residing at mild high altitude. The ODI reflects different clinical characteristics associated with OSAHS from a new perspective. Combining ODI monitoring with simple sleep screening equipment can provide a more accurate prediction and evaluation of OSAHS for medical institutions with limited resources in high-altitude areas.

Apnea can occur during both REM and NREM sleep. Average oxygen saturation during REM and NREM sleep differ according to the severity of sleep breathing disorders (21). Apnea is more common in NREM sleep and more severe in REM sleep (21). Previous research has reported that oxygen saturation does not differ significantly between patients with mild or moderate OSAHS living at sea level during REM and NREM sleep (21). Here, we discovered notable variations in MSpO_2_ values within the AHI <5/h group. Specifically, MSpO_2_ levels averaged at 90.41 ± 4.27, showing a significant difference between wakefulness (91.18 ± 4.61) and REM sleep (90.36 ± 5.11) (*p* < 0.01). However, the difference between REM and NREM sleep stages was not statistically significant (*p* = 0.16). Conversely, in the overall population of OSAHS patients, a progressive decrease in MSpO_2_ was observed from wakefulness to NREM sleep, and further into REM sleep (*p* < 0.01). Interestingly, patients with mild to moderate OSAHS exhibited significantly higher MSpO_2_ levels during REM sleep compared to NREM sleep (*p* < 0.01 for both groups). This trend was distinct from that observed in OSAHS patients who do not reside at mild high altitudes. Our findings therefore provide further evidence for differences in average oxygen saturation during REM and NREM sleep in patients with OSAHS and demonstrate that the severity of the disease is linked to sleep stages.

This study highlights the unique challenges and considerations of high-altitude environments. The impact of mild high altitude upon OSAHS severity and nocturnal hypoxia underscores the importance of tailored treatment approaches for patients in these regions. The evaluation of hypoxemia severity thresholds in high-altitude areas provides valuable clinical guidance for healthcare professionals assessing the severity of hypoxemia in OSAHS patients. Our study findings are of particular importance for improving OSAHS patient outcomes and quality of life.

The present study has some limitations. Firstly, it was a cross-sectional study conducted at a single center, which may have introduced selection bias. Secondly, conducting only first-night PSGs may have introduced bias due to poor sleep or altered sleep physiology caused by first-night effects. While each PSG report was considered adequate, it’s important to note that sleep efficiency can be influenced by first-night effects, impacting sleep quality and physiology. Thirdly, our patient cohort utilized the Alice-6 LDx, while healthy volunteers used the CONTEC RS01. Unfortunately, we did not directly compare the performance of these kits within the same subjects. Despite inherent variations, our study independently analyzed sleep patterns in each cohort using the specified kits. It is important to note that, as part of our research group, we have previously confirmed RS01’s reported consistency with Alice-6 (16). Fourth, all study subjects were exclusively from high-altitude areas. Additionally, for sea level data, we relied on published information. While this approach allowed us to gather relevant data, it introduces a potential limitation as the sea level data were not directly measured within our study cohort. Lastly, the relatively small number of non-OSAHS study participants led to a significant difference in group sizes between the OSAHS and non-OSAHS groups. Future clinical research is warranted to address these limitations.

## 5 Conclusion

This study has revealed important insights into the severity and clinical management of OSAHS. They suggest that patients, who reside in high-altitude areas, with an AHI score of <5/h should not be considered non-urgent, as they may require treatment due to the high risk of nocturnal hypoxia. The ODI has demonstrated that it can accurately differentiate OSAHS severity, making it a valuable diagnostic tool for healthcare professionals. Moreover, the associations between sleep stages and OSAHS severity highlights the importance of monitoring sleep stages in patients with OSAHS. Finally, the evaluation of hypoxemia severity thresholds in OSAHS patients living at mild high altitude provides valuable clinical guidelines for assessing their severity of hypoxemia. Overall, our findings contribute to a better understanding of OSAHS and provide important implications for the clinical management and treatment of this disease.

## Data availability statement

The raw data supporting the conclusions of this article will be made available by the authors, without undue reservation.

## Ethics statement

The studies involving humans were approved by the Qinghai Red Cross Hospital Institutional Review Board. The studies were conducted in accordance with the local legislation and institutional requirements. The participants provided their written informed consent to participate in this study.

## Author contributions

The study was conceived by LH. LH and KP designed the study. LH, KP, QB, SG, CD, LF, ZC, CR, HP, and ZM performed the experiments and data collection. The paper was written by LH. All authors contributed to the article and approved the submitted version.

## References

[1] De Backer W. Obstructive sleep apnea/hypopnea syndrome. Panminerva Med. (2013) 55:191–5. PMID: 23676959

[2] Memon J Manganaro SN. Obstructive sleep-disordered breathing. Treasure Island, FL: StatPearls Publishing (2022). 28722938

[3] Benjafield AV Ayas NT Eastwood PR Heinzer R Ip MSM Morrell MJ. Estimation of the global prevalence and burden of obstructive sleep apnoea: a literature-based analysis. Lancet Respir Med. (2019) 7:687–98. doi: 10.1016/S2213-2600(19)30198-5, PMID: 31300334 PMC7007763

[4] Sankri-Tarbichi AG. Obstructive sleep apnea-hypopnea syndrome: etiology and diagnosis. Avicenna J Med. (2012) 2:3–8. doi: 10.4103/2231-0770.94803, PMID: 23210013 PMC3507069

[5] Jennum P Riha RL. Epidemiology of sleep apnoea/hypopnoea syndrome and sleep-disordered breathing. Eur Respir J. (2009) 33:907–14. doi: 10.1183/09031936.00180108 19336593

[6] Mbata G Chukwuka J. Obstructive sleep apnea hypopnea syndrome. Ann Med Health Sci Res. (2012) 2:74–7. doi: 10.4103/2141-9248.96943, PMID: 23209996 PMC3507119

[7] Rashid NH Zaghi S Scapuccin M Camacho M Certal V Capasso R. The value of oxygen desaturation index for diagnosing obstructive sleep apnea: a systematic review. Laryngoscope. (2021) 131:440–7. doi: 10.1002/lary.28663, PMID: 32333683

[8] Wang TT Huang SX Zhang XM Zhang XW Luo YX. The relationship between oxygen saturation and related respiratory events in patients with obstructive sleep apnea-hypopnea syndrome. Lin Chuang Er Bi Yan Hou Tou Jing Wai Ke Za Zhi. (2017) 31:170–3. doi: 10.13201/j.issn.1001-1781.2017.03.002 29871216

[9] Wang L Wei DH Zhang J Cao J. Time under 90% oxygen saturation and systemic hypertension in patients with obstructive sleep apnea syndrome. Nat Sci Sleep. (2022) 14:2123–32. doi: 10.2147/NSS.S388238, PMID: 36474481 PMC9719713

[10] Liu P Chen Q Yuan F Zhang Q Zhang X Xue C. Clinical predictors of mixed apneas in patients with obstructive sleep apnea (OSA). Nat Sci Sleep. (2022) 14:373–80. doi: 10.2147/NSS.S351946, PMID: 35280432 PMC8906897

[11] Latshang TD Bloch KE Lynm C Livingston EH. Traveling to high altitude when you have sleep apnea. JAMA. (2012) 308:2418. doi: 10.1001/jama.2012.4097 23232901

[12] Pataka A Riha RL. The obstructive sleep apnoea/hypopnoea syndrome—an overview. Respir Med CME. (2009) 2:111–7. doi: 10.1016/j.rmedc.2009.03.001

[13] Stavrou VT Astara K Tourlakopoulos KN Papayianni E Boutlas S Vavougios GD. Obstructive sleep apnea syndrome: the effect of acute and chronic responses of exercise. Front Med. (2021) 8:806924. doi: 10.3389/fmed.2021.806924, PMID: 35004785 PMC8738168

[14] Myrzaakhmatova AK. Obstructive sleep apnea at high altitude. Ter Arkh. (2017) 89:103–6. doi: 10.17116/terarkh2017891103-106 28635906

[15] Huang L. High altitude medicine in China in the 21st century: opportunities and challenges. Mil Med Res. (2014) 1:17. doi: 10.1186/2054-9369-1-17 25937936 PMC4416354

[16] Pang Huaixia HL Zhen M Weizhen S Xiangrong H Chunmei C Baoliang Y. The diagnostic value of watch sleep monitoring in OSAHS at high altitude. World J Sleep Med. (2019) 6:1667–70. doi: 10.3969/j.issn.2095-7130.2019.12.011

[17] Wood EH. Normal oxygen saturation of arterial blood during inhalation of air and oxygen. J Appl Physiol. (1949) 1:567–74. doi: 10.1152/jappl.1949.1.8.567 18111529

[18] Radhakrishnan S Nair SG. Analysis of parameters affecting blood oxygen saturation and modeling of fuzzy logic system for inspired oxygen prediction. Comput Methods Programs Biomed. (2019) 176:43–9. doi: 10.1016/j.cmpb.2019.04.014 31200910

[19] Muaña D Oxygen saturation (medicine): National Jewish Health; (2009) Available at: https://www.scribd.com/document/360678527/Oxygen-saturation-medicine-pdf#

[20] Gries RE Brooks LJ. Normal oxyhemoglobin saturation during sleep. How low does it go? Chest. (1996) 110:1489–92. doi: 10.1378/chest.110.6.1489 8989066

[21] Choi E Park DH Yu JH Ryu SH Ha JH. The severity of sleep disordered breathing induces different decrease in the oxygen saturation during rapid eye movement and non-rapid eye movement sleep. Psychiatry Investig. (2016) 13:652–8. doi: 10.4306/pi.2016.13.6.652, PMID: 27909457 PMC5128354

[22] Zhao MM Zhang XL. Diagnosis and treatment of obstructive sleep apnea hypopnea syndrome. Zhonghua Yi Xue Za Zhi. (2012) 92:1228–30. PMID: 22883056

[23] Chinese Medical Association CMAJ, Chinese Medical Association General Practice Branch, Chinese Medical Association Respiratory Medicine Branch Sleep Respiratory Disorders Group, Chinese Medical Association “Chinese Journal of General Practitioners” Editorial Board, Basic Respiratory System Diagnosis and Treatment Guidelines Writing expert group. Guideline for primary care of adult obstructive sleep apnea. Chin J Gen Pract. (2018) 2019:30–5. doi: 10.3760/cma.j.issn.1671-7368.2019.01.007.

[24] Peng BG Lai YQ Lei HJ Zhang N Wang X. Strategies in the clinical diagnosis and surgical treatment of OSAHS with multilevel obstruction. J Int Med Res. (2019) 47:1533–43. doi: 10.1177/0300060518822209, PMID: 30966830 PMC6460628

